# Has increased nursing competence in the ambulance services impacted on pre-hospital assessment and interventions in severe traumatic brain-injured patients?

**DOI:** 10.1186/1757-7241-22-20

**Published:** 2014-03-19

**Authors:** Ann-Charlotte Falk, Annika Alm, Veronica Lindström

**Affiliations:** 1 Karolinska Institutet, Department of Neurobiology, Care Sciences and Society, Karolinska Institutet, Alfred Nobels Allé 23, III, 141 83 Huddinge, Stockholm, Sweden; 2 Karolinska Institutet, Department of Clinical Science and Education, Södersjukhuset, Academic EMS in Stockholm, Stockholm, Sweden

**Keywords:** Pre-hospital management, Competence, Patient outcome

## Abstract

**Objective:**

Trauma is one of the most common causes of morbidity and mortality in modern society, and traumatic brain injuries (TBI) are the single leading cause of mortality among young adults. Pre-hospital acute care management has developed during recent years and guidelines have shown positive effects on the pre-hospital treatment and outcome for patients with severe traumatic brain injury. However, reports of impacts on improved nursing competence in the ambulance services are scarce. Therefore, the aim of this study was to investigate if increased nursing competence level has had an impact on pre-hospital assessment and interventions in severe traumatic brain-injured patients in the ambulance services.

**Method:**

A retrospective study was conducted. It included all severe TBI patients (>15 years of age) with a Glasgow Coma Score (GCS) of less than eight measured on admission to a level one trauma centre hospital, and requiring intensive care (ICU) during the years 2000–2009.

**Results:**

651 patients were included, and between the years 2000–2005, 395 (60.7%) severe TBI patients were injured, while during 2006–2009, there were 256 (39.3%) patients. The performed assessment and interventions made at the scene of the injury and the mortality in hospital showed no significant difference between the two groups. However, the assessment of saturation was measured more frequently and length of stay in the ICU was significantly less in the group of TBI patients treated between 2006–2009.

**Conclusion:**

Greater competence of the ambulance personnel may result in better assessment of patient needs, but showed no impact on performed pre-hospital interventions or hospital mortality.

## Introduction

Trauma is one of the most common causes of morbidity and mortality in modern society, and traumatic brain injuries (TBI) are the single leading cause of mortality among young adults [1]. In the Nordic region, the mortality rate is 12.6/100 000 per year (male/female 18.8/6.4) [2], with the lowest median death rate in Sweden (9.5/100 000), compared to Norway (10.4), Denmark (11.5) and Finland (21.2). The acute management of patients with a TBI, both pre-hospital and in-hospital, has developed during the last 15 years, and evidence-based guidelines have been published [3-8]. According to the guidelines, the goal of the acute management is to identify patients in need of acute intervention as early as possible to prevent secondary brain injuries due to hypoxia and/or hypotension, and thereby minimize the impact of long-term disabilities [3-7]. Variables that would predict long-term outcome after TBI have been presented, such as the Glasgow Coma Score (GCS), pupil reaction, age at injury and head-computed (CT) scan findings [9-11]. However, the majority of these variables are measured on arrival in the primary hospital, not in the pre-hospital setting [12]. This happens despite the fact that the personnel in the Emergency Medical System (EMS) are the first health care providers and make the first assessment and perform interventions at the scene of an injury. The EMS personnel have varied education, skills and qualifications [12-14], and previous studies have shown that the pre-hospital treatment of patients may differ depending on the profession of the health care staff that first treat them, e.g. the time at the scene may increase with a physician-based EMS, and a paramedic-based EMS may cause lack of proper pre-hospital airway management [15,16]. As for evaluation of nursing care, the findings from Naylor et al. show that nursing care is both central and essential to the delivery of high quality care in different health care settings [17]. In the hospital setting, the result of Aiken et al. showed a positive link between nurses’ educational level and decreased mortality rates and failure to rescue [18]. However, reports of impact on improved nursing competence in the ambulance services are scarce. In the year 2005, there was a change in competence in the ambulance services in Sweden due to regulation from the National Board of Health. Every ambulance was then manned by one emergency medical technician (EMT) and one registered nurse(RN) with advanced life support competence in contrast to two emergency medical technicians (EMT) with basic life support knowledge. This change to ensure higher nursing competence may theoretically have improved the ability to perform advanced care and treatment during ambulance care and may have an effect on outcome for the TBI patient. Therefore, the aim of this study is to investigate if increased competence has impacted on pre-hospital assessment and interventions in severe traumatic brain-injured patients treated by the ambulance services.

## Method

A retrospective observational study was conducted at a university hospital in Sweden during the years 2000–2009. The Regional Ethical Review board approved the study (Dnr: 2010/192).

### Setting

The EMS and the university hospital (a level one trauma centre) cover approximately 2.1 million inhabitants in the Stockholm area. The transport-time in the region is less than 60 minutes. The incidence of brain injury in the region during the study period was 126–160 per 100 000 inhabitants/year [19]. The regional County Council is responsible for the EMS, and during the study period the service was provided by the one organization within the county and three private companies contracted by the County Council. During the study period the ambulance fleet consisted of 55 ambulances. Two anaesthesia-nurse manned emergency cars and one physician-manned helicopter were available to assist the ambulance personnel.

Prior to the change of competence in ambulances, an ambulance crew consisted of two EMTs, who were re-certified every other year with knowledge of basic life support (BLS). After September 2005 the ambulance crew consisted of one EMT and one RN with at least three years of standard RN training, who were re-certified every other year. The RNs have the knowledge and skills for advanced life support (ALS) and can give intravenous drugs, depending on the patient’s condition [20].

Overall, there were no pervasive changes regarding the specific care of TBI patients in the EMS during the study period.

### Patients and data collection

Data were collected from the Brain Trauma injury database between 2000–2009, including all adult (> 15 years) severe TBI patients with GCS less than eight measured on admission, requiring intensive care. No patients were excluded.

### Measures

Data collected from the database was: in-hospital demographic variables (age, gender, mechanism and type of injury, GCS) and pre-hospital documentation of initial GCS, performed assessment of vital signs and interventions concerning airway and circulation. The GCS measures the level of consciousness by scoring: eye opening, motor response and verbal response. The scores vary between 3–8 (severe brain injury), 9–13 (moderate brain injury) and 14–15 (mild brain injury) [21]. For this study, severe TBI was defined as a patient with GCS less than eight on admission to hospital.

### Outcome

Outcome measures were: in-hospital mortality and length in intensive care unit (ICU).

### Statistics

Descriptive statistical procedures were computed using the SPSS version 20.0 for Windows, Chicago, IL, USA. The data was divided into two groups, before and after 2005, and a group comparison was made. Categorical variables were compared by means of Fisher’s exact two-tailed test or Pearson chi-square tests. Probability below 0.05 was accepted as statistically significant. No power calculation was performed due to the non-experimental design.

## Results

**Figure 1:**
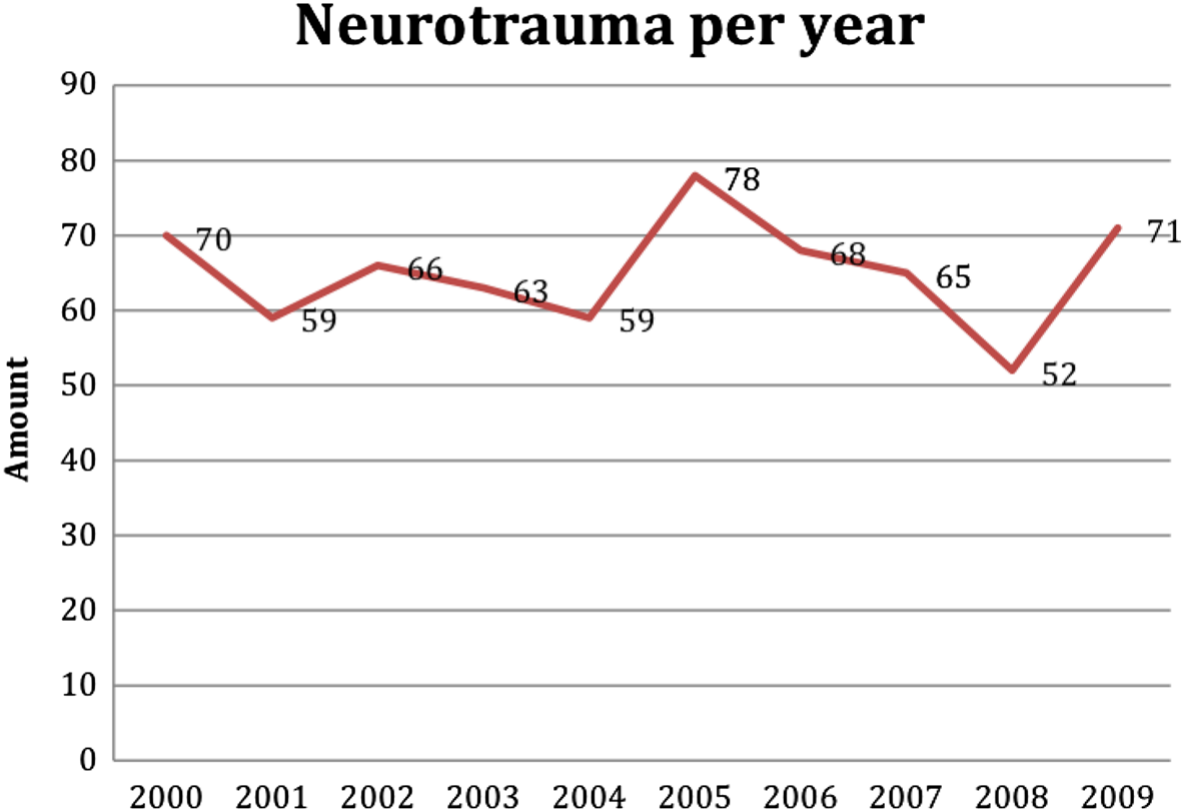
The frequency of patients with TBI.

For the study, 651 patients were collected from the trauma registry. Of these patients, 395 (60.7%) were injured between the years 2000–2005 and 256 (39.3%) during the years 2006–2009. No exclusions were made. It was found that 84 per cent of the patients were transported by ambulance and 16 per cent were transported by helicopter. The frequency of included patients with TBI per year is displayed in Figure 1.

In the entire group of patients there was a majority of men (n = 503, 77%) with a TBI compared to 148 (23%) females with mean age of 48 years (range 15–94, median 52) (Table 1).

**Table 1:** Demographic data for all patients n = 651 with a severe traumatic brain injury

	**Year 2000-2005**	**Year 2006-2009**
	**n (%)**	**n (%)**
**Total patients**	395	256
**Mean age**	47.8	49.2
**Mechanism of trauma**		
Blunt trauma	379 (96)	236 (92)
Penetrating trauma	16 (4)	20 (8)
**Injury characteristics**		
Brain injury	330 (83)	185 (75)
Brain injury including other injuries	66 (16)	70 (27)
**External cause of injury**		
Fall < 3 m	228 (58)	158 (62)
Fall > 3 m	21 (5)	22 (9)
Road and traffic accident	87 (22)	55 (21)
Other	59 (15)	21 (8)
**GCS at scene**		
3-8	232 (58)	150 (58)
9-12	97 (25)	57 (23)
13-15	64 (16)	43 (17)
Missing	2 (1)	5 (2)

The most common cause of trauma among all patients were falls from less than three meters (n = 386, 58%) and GCS at the scene showed that out of all patients, 42 per cent had a GCS score over 8 at the scene of the accident. No difference was found between the two study periods and the distribution of GCS scores on the scene of the injury or on admission to hospital.

### The performed assessment and interventions

The performed assessment and interventions made at the scene of the injury, showed no significant difference between the two groups (Table 2). The assessment of saturation was measured more frequently during the years after 2006 (57% vs. 67%). No difference was between the two groups whether or not the ambulance personnel had assistance from anaesthesia-nurse or the physician-manned helicopter.

**Table 2:** Description of documented assessment and interventions in all patients n = 651 with a severe traumatic brain injury

	**Year 2000-2005**	**Year 2006-2009**
	**395 n (%)**	**256 n (%)**
**Assessments**		
Airway (clear airway)	247 (63)	132 (51)
Breathing (Saturation)	254 (57)	171 (67)
Circulation (BP)	264 (67)	161 (63)
Neurology (GCS)	385 (97)	246 (96)
Documentation of secondary insults (Sat < 90, BP <90)	80 (21)	46 (18)
**Interventions**		
Intubation on scene	71 (18)	49 (19)
I.V. fluid	124 (31)	73 (29)
Sedation	239 (61)	158 (61)
**Direct transportation to level 1 trauma centre**	201 (51)	141 (55)
**Outcome**		
**Length of ICU stay (mean)**	11.16 days	8.82 days
**In hospital mortality**	120 (30)	75 (29)
**GOS at 3 months**		
Favourable (GOS 4–5)	56 (14)	15 (6)
Unfavourable (GOS 1–3)	339 (86)	241 (94)

Regarding the primary outcome measured by mortality in hospital; there were no statistically significant differences (χ^2^ = .087, *df* =1, *p* = 0.77) between the two groups.

However, the mean length of stay in the ICU was significantly less in the group of patients during 2006–2009 (*p=. 0001*).

## Discussion

The ambulance service in Sweden has developed from an organization mainly transporting patients to hospital to an organization with advanced care and medical treatment [22].

Our result indicates that increased competence of RNs in the ambulances may have had an impact on performed assessments. The fact that assessment of saturation was more frequently documented could be the result of increased overall competence. This is supported by the result of Laudermilch et al. who showed that complete documentation of physiological data decreases mortality to 4.5% v.s 10.3% compared to those with incomplete documentation in the EMS [23]. This could reduce the number of secondary insults at the scene of the injury, if the assessment leads to interventions such as administration of I.V. fluid, oxygen, management of airway and identifying optimal level of care. However, the impact on primary outcome, mortality in hospital, showed no significant difference before and after RNs was regulated to work in the ambulances. The outcome measure GOS showed a decrease after 2006 (14% vs. 6%). This could be related to the fact that more patients (in the group after 2005) had other injuries as well as a severe brain injury.

The lack of positive results could be due to a number of reasons; the fact that the used measures were not applicable to measuring quality of nursing care is one. As reported by Naylor et al. health status, quality of life, and patients’ or relatives’ experiences may be used to measure quality of nursing care in a health care setting [17]. However, in the ambulance services, there might be other measures that would capture the quality of nursing. To further study indicators of quality in different nursing settings such as RNs’ ability in early identification of the optimal level of care for the patient would contribute to the debate on the need for higher competence among nurses in the EMS.

The fact that the outcome measures used in this study were not measured in immediate contact to the EMS care but during the hospital stay should also be accounted for. The length of ICU stay may have shortened after 2005 when the RNs were regulated to work in the ambulances. One reason for the shorter hospital stay could be improved treatment and care in hospital; another reason could be the development of improved care in the EMS, as reported by Rudehill and Härtl [24,25].

Another reason could be the used study population; a patient with a severe TBI (GCS < 8 on admission to hospital) may actually not be a challenge for the ambulance personnel as assessing patient needs, immediate care and treatment are continuously trained for. The results of this study show that the distribution of GCS score both on the scene of the injury and on admission to hospital did not differ between the two study periods, which could mean that increased competence did not have an impact on the primary injury. This may point to the need for awareness of the difficulties in early identification of severe TBI and the potential for optimal management [11]. The fact that the majority of patients fell less than three meters may indicate that falling from standing is more dangerous in regard to a traumatic brain injury than road and traffic accidents in Sweden. Consequently, a revision of existing guidelines as reported by Dunning et al. should be considered [26].

The fact that the transportation time in the region is less than 60 minutes could also be another explanation for the low impact on the interventions. However, further studies are needed to investigate the true impact on EMS competence on patient outcome and to explore possible indicators of nursing care in the ambulance.

There are some limitations in our study to be considered when evaluating our results. The retrospective methodology gives no possibility to randomize patients to be cared for by a nurse or not, and variables may not be documented in the registry due to lack of documentation in the ambulance medical record. Another limitation is that our result can be influenced by changes in unknown external factors, such as the availability of ambulances and helicopters may have occurred throughout the study period.

The differences in the number of patients between the study groups could also have had an impact on the result. The best way to evaluate EMS care would be to measure outcome in the immediate contact to the EMS care but that was not possible during this period. For the future, continuous evaluation of the EMS care to improve patient outcome should be the main focus.

## Conclusion

Implementing more competent personnel in the ambulance may have resulted in better assessment of patient needs but showed no impact on performed pre-hospital interventions or hospital mortality.

## Competing interests

There are no conflicts of interest.

## Authors’ contributions

ACF and VL designed and performed the study. AA helped the draft of the manuscript. All authors read and approved the final manuscript.
